# Supplementary material for: Polyketide synthase gene clusters in the ericoid mycorrhizal fungus Oidiodendron maius: genome-wide prediction, expression profiling, and functional characterization of knockout mutants
Source: Front Plant Sci. 2026 Sep 16;17:1924430. doi: 10.3389/fpls.2026.1924430 (PMC13623903; doi:10.3389/fpls.2026.1924430)
Supplement: Supplementary file 1 [file SupplementaryFile1.zip › Supplementary Materials/Supplementary Folder 1.zip!/Oidma1_AssemblyScaffolds_Repeatmasked/knownclusterblast/cluster7/jgi.p_Oidma1_111078_mibig_hits.html]

| MIBiG Protein | Description | MIBiG Cluster | MiBiG Product | % ID | % Coverage | BLAST Score | E-value |
| --- | --- | --- | --- | --- | --- | --- | --- |
| BAE95592.1 | putative\_dehydratase | BGC0000703 | Kanamycin\_biosynthetic\_gene\_cluster | 32 | 96.3446475196 | 186 | 7.2e-47 |
| ADC45548.1 | mandelate\_racemase | BGC0000093 | Meilingmycin\_biosynthetic\_gene\_cluster | 27 | 91.9060052219 | 103 | 6.1e-22 |
| BAE95548.1 | mandelate\_racemase/muconate\_lactonizing\_enzyme | BGC0000703 | Kanamycin\_biosynthetic\_gene\_cluster | 26 | 89.5561357702 | 82 | 8.6e-16 |
